# Zoonotic Helminths in the Southern Peruvian Altiplano: A Four-Year Sero-Epidemiological Study and One Health Policy Implications

**DOI:** 10.3390/ijerph23010080

**Published:** 2026-01-06

**Authors:** Polan Ferro-Gonzales, Pompeyo Ferro, Patricia Matilde Huallpa Quispe, Euclides Ticona, Jorge Bautista Nuñez, Ana Lucia Ferró-Gonzáles

**Affiliations:** 1 Departamento Académico de Ingeniería Económica, Facultad de Ingeniería Económica, Universidad Nacional del Altiplano, Puno 21001, Peru; 2 Instituto de Investigación del Altiplano, Universidad Nacional del Altiplano, Puno 21001, Peru; 3 Departamento Académico de Ciencias Naturales y Aplicadas, Facultad de Ciencias Naturales y Aplicadas, Universidad Nacional Intercultural Fabiola Salazar Leguía de Bagua, Bagua 01721, Peru; fferro@unibagua.edu.pe (P.F.); eticonac@unibagua.edu.pe (E.T.); 4 Departamento Académico de Contabilidad, Facultad de Ciencias Empresariales, Universidad Nacional de Cañete, San Vicente de Cañete 15800, Peru; phuallpa@undc.edu.pe; 5 Departamento Académico de Ingenierías, Facultad de Ingenierías, Universidad Nacional Intercultural Fabiola Salazar Leguía de Bagua, Bagua 01721, Peru; jbautista@unibagua.edu.pe; 6 Departamento Académico de Gestión y Ciencias Sociales, Facultad de Gestión y Emprendimiento Empresarial, Universidad Nacional de Juliaca, Juliaca 21101, Peru; al.ferrog@unaj.edu.pe

**Keywords:** echinococcosis, fasciolosis, taeniosis/cysticercosis, one health, zoonoses, Peru

## Abstract

We assessed the prevalence of three helminthic zoonoses—echinococcosis, fasciolosis and the taeniosis/cysticercosis complex—among residents of the Chucuito Health Network (Puno Health Region, Peru) over four years (2018–2021). Sera (*n* = 910) were analysed by ELISA to detect pathogen-specific antibodies, following national protocols. Echinococcosis predominated, whereas fasciolosis and taeniosis/cysticercosis occurred at comparatively low levels. Prevalence ranged from 4.4–9.2% for echinococcosis, 1.1–4.9% for fasciolosis, and 1.1–2.7% for taeniosis/cysticercosis across the four years. Prevalence varied significantly between years, with a notable upsurge in echinococcosis in 2021. These findings underscore the need for integrated control and prevention measures grounded in a One Health framework that recognises the interconnections between human, animal and environmental health. Priority actions include strengthened health education programmes, improved hygiene and sanitation practices, and enhanced rural health infrastructure, alongside coordinated epidemiological surveillance and environmental management. Such measures are essential to mitigate the burden of zoonotic disease in vulnerable high-Andean communities.

## 1. Introduction

In recent years, the global community has devoted greater attention to zoonoses and other communicable diseases that affect both humans and animals, many of which have long been classified as neglected diseases [1]. These infections predominantly affect pastoral rural areas where inadequate sanitation, low educational attainment, and the absence of prevention and control programmes facilitate their spread [2]. This situation underscores the need to improve our understanding of zoonotic epidemiology, transmission mechanisms, diagnosis, prevention and control [3].

Zoonoses exert a wide range of deleterious effects, and their high incidence continues to drive morbidity and mortality in both humans and animals. These diseases are naturally transmitted to humans from domestic or wild animals [3], and climate change is a major driver of their emergence and resurgence [4,5].

More than 150 zoonoses have been described, and many occur virtually worldwide [6]. At least 800 pathogens cause zoonotic disease, and approximately 60% of all humans’ pathogens are zoonotic, underscoring their relevance to human development [7]. Key helminth-mediated zoonoses include nematodes (e.g., trichinellosis), cestodes (e.g., cysticercosis, echinococcosis) and trematodes (e.g., schistosomosis, fasciolosis, often termed fascioliasis in the human health literature), among others [8]. Hydatidosis (echinococcosis) caused by a cestodes of the *Echinococcus granulosus* sensu lato complex, comprising several species/strains, remains a significant public-health problem in South America and worldwide [9]. The parasite’s life-cycle involves herbivorous intermediate hosts and canine definitive hosts, while humans act as accidental intermediate hosts who ingest eggs via contaminated food, water, or direct contact with infected dogs [10]. Echinococcosis exhibits a high human case-fatality rate and imposes substantial economic losses through reduced productivity and the cost of treatment and hospitalization [11].

Fasciolosis, transmitted by *Fasciola* spp. via lymnaeid snails, is widespread in Peru and globally owing to the parasite’s capacity to colonise diverse snail hosts and adapt to varied climates. It is the vector-borne disease with the broadest latitudinal, longitudinal, and altitudinal distribution [12].

*Taenia solium* taeniosis/cysticercosis complex (also termed taeniasis/cysticercosis in WHO documents), a human intestinal infection, and its larval form (cysticercosis) in pigs and humans, can target the central nervous system (neurocysticercosis) [13] and provoke seizures, headaches, dizziness, and, if untreated, mortality [14]. Neurocysticercosis is a leading cause of symptomatic epilepsy in developing nations.

Although water-borne zoonoses have been widely documented over the last three decades [15], reporting remains limited in Peru. Prevalence estimates in humans include cysticercosis 3%, hydatidosis 5%, and fasciolosis 11% [16], with heterogeneous frequencies across region [17].

The One Health approach, which recognises the interdependence of human, animal, and environmental health, is essential for the effective management of zoonoses. Approximately 75% of emerging pathogens are zoonotic, with wildlife acting as the principal reservoir [18,19]. The One Health framework fosters cross-sector collaboration, enhancing surveillance and response [20]. The COVID-19 pandemic vividly illustrates the need for integrated measures to prevent animal diseases spilling over into human populations.

Despite progress, implementation of One Health remains incomplete, particularly regarding the integration of environmental science with human and veterinary medicine [21]. Urbanisation and climate change amplify zoonotic risks and demand adaptive strategies [22]. Resource constraints notwithstanding, it is increasingly recognised that the social ecology of zoonoses must be addressed more comprehensively [23].

In Peru, fasciolosis is highly prevalent in ruminants, with national estimates indicating frequent liver condemnation in cattle and sheep due to *Fasciola hepatica* [24]. Cystic echinococcosis is widespread in sheep, cattle and camelids, with dogs acting as definitive hosts and maintaining intense rural transmission cycles [25,26,27]. *Taenia solium* cysticercosis in pigs is focal yet substantial in several endemic rural areas, where high porcine seroprevalence and strong spatial clustering around human tapeworm carriers have been reported [28,29,30,31].

The Ministry of Health of Peru now requires that zoonotic disease surveillance adopts a One Health perspective [32]. This research aims to screen for three zoonotic diseases in the human population, considering that the ecoepidemiology of zoonoses is often oversimplified and therefore limiting ourselves to host–pathogen interactions [33].

## 2. Materials and Methods

### 2.1. Study Area

**Figure 1:**
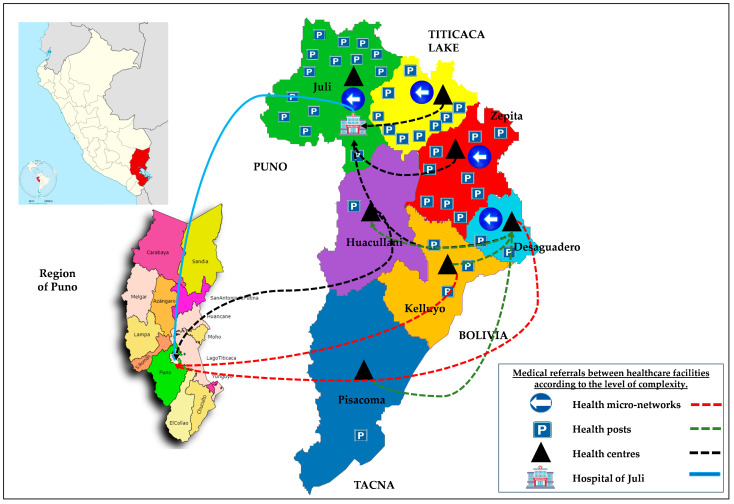
Geographical location of the Chucuito Health Network in the southern Peruvian Altiplano.

The research was conducted within the Chucuito Health Network (CHN) in the Puno Health Region of south-eastern Peru (Figure 1) between January 2018 and December 2021. The CHN comprises thirteen predominantly rural districts characterised by intensive camelid, cattle and sheep husbandry—conditions that heighten the risk of zoonotic transmission. The investigation formed part of Budgetary Programme 010 “Metaxenic and Zoonotic Diseases” and complied fully with Ministry of Health regulations [32].

The CHN lies at 3800–4200 m above sea level on the southern Peruvian Altiplano, with a cold semi-arid climate, mean annual temperature ~7–8 °C and marked wet (November–March) and dry (April–October) seasons. The network serves approximately 130,000–140,000 inhabitants across thirteen rural districts. Livelihoods are dominated by extensive sheep and cattle rearing and the keeping of camelids (llamas, alpacas) and backyard pigs, with free-roaming dogs commonly present around households and grazing areas.

### 2.2. Study Design and Sampling

We conducted a cross-sectional, observational study nested within the routine serological screening activities of the Chucuito Health Network. All individuals attending participating primary health centres during the study period and for whom sufficient serum was available were eligible for inclusion. Sampling was therefore by consecutive inclusion rather than probabilistic randomisation. The minimum required sample size (*n* = 384) to detect a prevalence of 5% with 95% confidence and 2% precision was exceeded, as 910 sera were ultimately analysed. Serum samples originated from individuals of all ages attending CHN health facilities for routine care or clinical complaints. The secondary anonymised dataset supplied by the Environmental Health Unit did not include personal identifiers or detailed age categories, precluding linkage of repeated visits. Because the anonymized dataset did not contain personal identifiers, we could not exclude repeated testing of some individuals; however, local programme staff indicated that most screenings are one-off, opportunistic tests rather than routine serial monitoring. Annual sample numbers and population denominators are summarised in Table 1.

### 2.3. Sample Collection

Venous blood samples were obtained from residents presenting to primary health centres within the CHN. Collections were performed by trained health personnel following Ministry of Health standard operating procedures and the Peruvian National Institute of Health procedures manual for the Serological Diagnosis of Parasitic Zoonoses [34]. Within two hours of collection, samples were centrifuged at 3000× *g* for 10 min; sera were refrigerated at 4 °C in the Environmental Health Unit until their weekly transfer—under cold chain—to the ISO-9001-certified Bioproject Laboratory in Puno.

### 2.4. Serological Testing

The ELISA (Enzyme-Linked Immunosorbent Assay) method was used for serological analysis of the samples. This immunoenzymatic test allows the detection of the presence of specific antibodies for echinococcosis, fasciolosis and the taeniosis/cysticercosis complex through the reaction between the antibodies present in the sample and the antigens adhered to the polystyrene surface. The ELISA test was selected for its sensitivity and specificity in the detection of these zoonotic diseases in rural contexts. Sera were screened for IgG antibodies against *Echinococcus granulosus*, Fasciola hepatica and the Taenia solium complex using commercial ELISA kits. These assays detect IgG and therefore reflect past exposure rather than acute infection.

Sera were tested using commercially available ELISA kits (RIDASCREEN^®^ *Echinococcus* IgG, RIDASCREEN^®^ *Fasciola* IgG, and RIDASCREEN^®^ *Taenia solium* IgG; R-Biopharm AG, Darmstadt, Germany), following the manufacturer’s instructions and the Peruvian National Institute of Health procedures manual for the Serological Diagnosis of Parasitic Zoonoses [34]. These kits employ recombinant or purified antigens bound to polystyrene plates to detect parasite-specific IgG.

### 2.5. Statistical Analysis

Statistical tests were performed to assess differences in prevalence between diseases and between years studied. First, data normality was checked using the Shapiro–Wilk test. Proportions were compared using Fisher’s exact tests (2 × 4 contingency tables for each pathogen across years). Where indicated, pairwise post hoc comparisons were conducted with Bonferroni-adjusted *p*-values. Principal Component Analysis (PCA) was used to explore patterns in annual prevalence across the three pathogens instead of temporal relationships. Marginal means were also calculated for each zoonotic disease.

## 3. Results

The total number of samples collected was 910, distributed in the following years: 182 in 2018, 168 in 2019, 376 in 2020 and 184 in 2021 (Table 1). The samples reflect the population assisted in the health centres of the CHN, which covers a range of rural communities exposed to risk conditions for the transmission of zoonoses.

**Figure 2:**
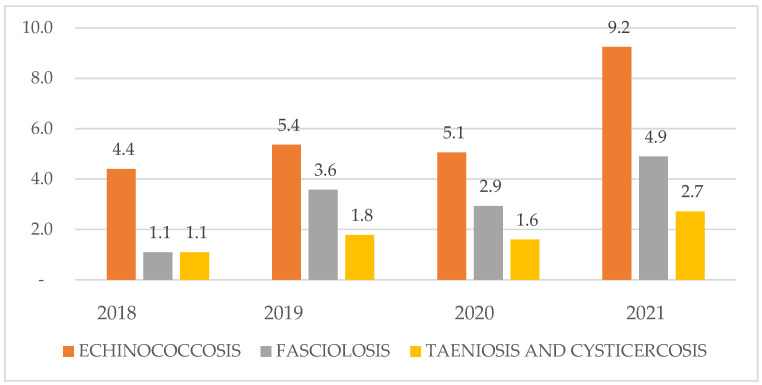
Annual seroprevalence of the three zoonotic diseases investigated in the Chucuito Health Network, 2018–2021.

- Echinococcosis showed the highest prevalence, peaking in 2021 (9.2%) compared with 2018–2020 (4.4–5.4%) (Figure 2).
- Fasciolosis remained comparatively low, rising from 1.1% in 2018 to 4.9% in 2021.
- The taeniosis/cysticercosis complex exhibited the lowest prevalence throughout, increasing modestly from 1.1% (2018) to 2.7% (2021).

### Marginal Means

**Figure 3:**
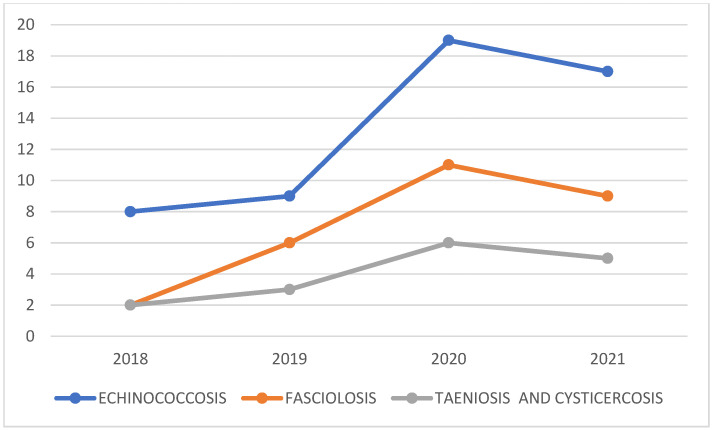
Marginal means of seroprevalence for the three zoonotic helminths in the Chucuito Health Network, 2018–2021.

The marginal means indicate that the overall prevalence of the three zoonotic diseases was broadly comparable during the study period (Figure 3).

From Table 1, at a significance level of <0.05; we found evidence that disease prevalence differed across the four survey years; post hoc comparisons identified year-on-year differences for each condition. Echinococcosis prevalence in 2021 (9.2%) was higher than in 2018–2020 (4.4–5.4%), and Fisher’s exact tests confirmed significant between-year differences for echinococcosis and fasciolosis, but not for taeniosis/cysticercosis.

**Figure 4:**
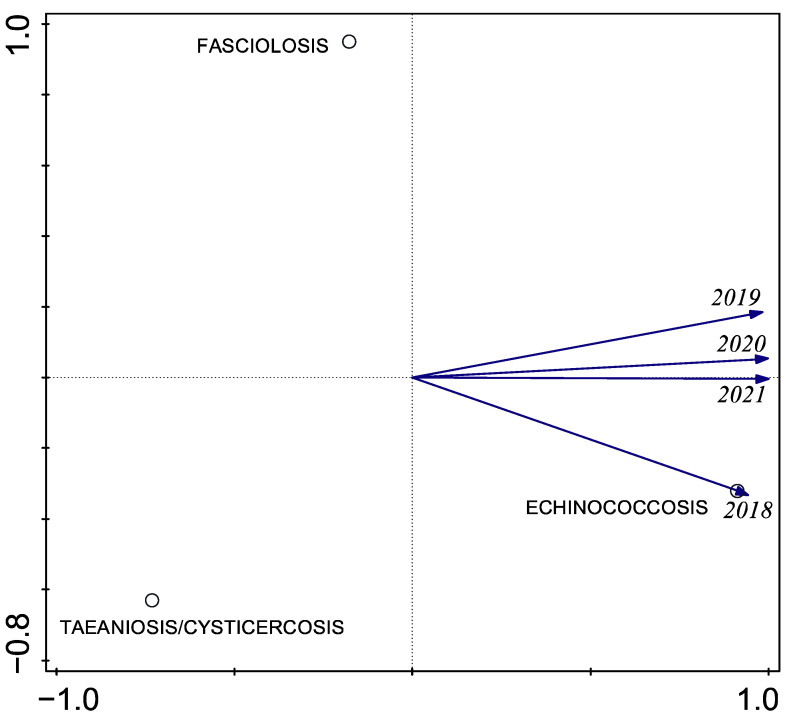
Principal component analysis (PCA) of annual prevalence of the three zoonotic helminths in the Chucuito Health Network, 2018–2021.

Using PCA, we identified a strong loading of echinococcosis on the first component in the final three years of sampling (Figure 4). This pattern is consistent with the descriptive observation that echinococcosis prevalence was higher in 2021 than in previous years, although formal trend testing with only four time points has limited power and should be interpreted with caution.

These results (Table 2) suggest that echinococcosis is becoming increasingly prevalent within the Chucuito Health Network, whereas fasciolosis and taeniosis/cysticercosis remain comparatively stable but warrant continued surveillance.

## 4. Discussion

Regarding echinococcosis, our observed prevalence (9.2% in 2021) exceeds that reported by Castillo Benancio [16] (5%), Lorca et al. [35] (2.5%) and García-Apaico et al. [36] (3.7%), yet remains below the markedly higher figures described for Cerro de Pasco (24.5%; Antitupa et al. [17]) among others [15,16]. We concur with Guerra and Ramirez [37] that echinococcosis is highly endemic in Andean Peru. Environmental and socio-economic heterogeneity—altitude, aridity, pastoral practice and limited veterinary oversight—probably underlie the inter-regional variation. Notably, most echinococcosis cases diagnosed in Lima originate in Andean migrants, predominantly young adults (73.5%; [17,38] being the most frequent in rural areas of Latin America [10]. Mathematical models suggest that sustained, multisectoral control would require at least two decades to eliminate transmission [11].

For fasciolosis, we identified a much lower prevalence (≤4.9%) than the 11% recorded by Castillo Benancio [16] and the 40.6% in Puno, 20% in Cajamarca and 15.4% in Arequipa reported by Antitupa et al. [17]—areas long recognised as endemic [39,40,41,42]. Our figures nevertheless exceed the 4.17% reported recently for other Puno districts [43]. Local abundance of *Lymnaea* spp. snails, flood-irrigated pasture and livestock density determines focal transmission, reinforcing the need for targeted environmental control and routine veterinary deworming [12].

The taeniosis/cysticercosis complex remained relatively uncommon (≈3%), a proportion comparable with rural Peru [16] and slightly above the Latin-American average of 2.3% [4]. Lower rates in Huánuco (1.9%) and Huancavelica (0.5%) [4] may reflect improved meat inspection and latrine coverage. Conversely, the very high prevalences reported for Cuzco (24%) and Andahuaylas (12%) underline persistent sanitation gaps [4] as well as the report of 3.3% in Ayacucho by Ayala et al. [44]. Enhanced community-based health education—shown to curb transmission elsewhere [45]—could further reduce the burden in Chucuito.

Overall, data on these three zoonoses remain scarce and geographically patchy [46]. Climate change, by altering vector and host ecology, is likely to exacerbate transmission [4,22,23]. Given that ~99% of endemic zoonoses arise in anthropogenic settings [33], an integrated One Health strategy is imperative [1]. Such an approach demands robust inter-ministerial governance, coordinated surveillance, joint mass-drug administration, environmental management and community engagement [18,20,21]. While regional and global studies suggest that climate variability may influence snail habitats and livestock management [47,48], robust evidence directly linking recent climatic trends to the human prevalence patterns observed in Peru remains limited. We therefore mention climate change as a potential contextual driver rather than a confirmed causal factor in this setting.

It is noteworthy that variations in disease notification may stem from differences in exposure to zoonotic agents, household hygiene practices, the reach of health education programmes, and the robustness of local health infrastructure. Pinpointing these determinants is indispensable for designing targeted interventions that can meaningfully lower incidence [49]. Our findings likewise underscore the need for sustained investigation into the socio-ecological drivers unique to southern Peru, as proposed by Desvars-larrive and Vogl [33]. A thorough understanding of these contextual factors will enable the development of measures tailored to local realities and thus more effectively reduce the zoonotic burden. We therefore advocate the roll-out of comprehensive health education initiatives and public-awareness campaigns aimed at improving hygiene and sanitation in rural communities, consistent with the recommendations of Villar Aguirre [49]. The ELISA kits detect IgG only and cannot distinguish acute from past infection, so our seroprevalence estimates should be interpreted as indicators of cumulative exposure.

In Peru, molecular studies have documented the circulation of several species within the *Echinococcus granulosus* sensu lato complex in high-Andean livestock and humans. Early work by Sanchez et al. [50] showed the presence of *E. granulosus* in multiple intermediate hosts, and more recent analyses from the Department of Puno have further characterised the species and genotypes involved. In a panel of 152 echinococcal cysts collected from sheep, cattle, pigs and South American camelids across ten localities, *E. granulosus* sensu stricto (G1 genotype) predominated in all host species, whereas *E. canadensis* (G7 genotype) was detected only in pigs and alpacas. This investigation also provided the first report of *E. granulosus* s.s. and *E. canadensis* infections in llamas and alpacas, respectively, in Peru, underscoring the complexity of multi-host transmission cycles in the southern highlands. When interpreted alongside our human sero-survey, these molecular data strengthen the evidence for intense, locally sustained transmission and highlight the need to tailor preventive programmes—including regular dog deworming, safe offal disposal, and the strategic use of livestock vaccination and chemotherapy—to the *E. granulosus* s.l. species and genotypes circulating in the southern Peruvian Altiplano.

This study has several limitations. First, it is based on a cross-sectional convenience sample of health-facility attendees rather than a population-based random sample, which may limit generalisability. Second, as we analysed an anonymised programme dataset without unique identifiers, we could not exclude repeated sampling of some individuals or stratify prevalence by age group. Third, the ELISA assays detect IgG only and cannot distinguish active from past infection. Fourth, we lacked individual-level covariates such as age, sex, occupation and animal contact, so we cannot model risk factors. These caveats should be considered when interpreting the findings. Finally, although the RIDASCREEN^®^ *Echinococcus* IgG, *T. solium* IgG and *Fasciola* IgG tests offer high analytical performance, the manufacturer’s manuals emphasise that ELISA alone is insufficient for definitive diagnosis. Cross-reactions with other parasitic infections can occur, and serological results should ideally be corroborated by additional methods such as imaging (e.g., ultrasound, CT) and clinical assessment. Our serological findings should therefore be interpreted as indicative of exposure rather than as a substitute for case-level diagnostic workup.

## 5. Conclusions

The present study leads to the following priority issues:

1. Marked heterogeneity in zoonotic burden

Echinococcosis remains disproportionately prevalent in the southern Peruvian Altiplano, whereas fasciolosis and taeniosis/cysticercosis occur at markedly lower levels. Tailored surveillance and control measures must therefore reflect the distinct epidemiology of each pathogen.

2. Recent upsurge in case numbers (2021)

All three diseases—most conspicuously echinococcosis—showed their highest observed prevalence in 2021. With only four annual surveys and modest case counts, this apparent increase should be regarded as a signal for further investigation rather than definitive evidence of a sustained upward trend.

3. Imperative for a One Health framework

The data reinforce the value of an integrated One Health strategy that acknowledges the inseparable links between human, animal and environmental health. Such an approach is pivotal for addressing the multifactorial dynamics that sustain zoonotic transmission in vulnerable high-altitude settings.

4. Priority interventions
  - Strengthen community health education to promote safe husbandry, meat inspection and hygiene practices;
  - Upgrade rural health infrastructure and laboratory capacity for timely diagnosis;
  - Implement targeted environmental control—for example, canine deworming and snail-habitat management—focused on the most prevalent zoonoses.
5. Research agenda

Future work should quantify the ecological, climatic and social determinants of transmission at fine spatial scale, thereby enabling precision public-health interventions. Longitudinal studies and participatory epidemiology will be essential for tracking trends, evaluating control programmes and ultimately reducing the zoonotic disease burden across the Andean region.

## Figures and Tables

**Table 1:** Population, sample size and seroprevalence of human echinococcosis, fasciolosis and Taenia solium taeniosis/cysticercosis in the Chucuito Health Network, 2018–2021.

Year	Population (Chucuito Province)	Sample Size (*n*)	Echinococcosis * *n* (%)	Fasciolosis * *n* (%)	Taeniosis/Cisticercosis * *n* (%)
2018	130,500	182	8 (4.4%)	2 (1.1%)	2 (1.1%)
2019	134,460	168	9 (5.4%)	6 (3.6%)	3 (1.8%)
2020	137,930	376	19 (5.1%)	11 (2.9%)	6 (1.6%)
2021	138,990	184	17 (9.2%)	9 (4.9%)	5 (2.7%)

Note: * Pairwise Fisher’s exact tests indicated significant differences between years for echinococcosis and fasciolosis (*p* < 0.05); taeniosis/cysticercosis differences were not statistically significant. Population figures correspond to annual estimates for Chucuito Province. Seroprevalence is expressed as the proportion of ELISA-positive sera among all samples tested for each zoonotic disease in a given year.

**Table 2:** Pearson correlation coefficients between annual prevalence values, calendar year and disease category for the three zoonotic helminths (*n* = 12 observations; four years × three diseases).

Correlations				
		Values	Years	Diseases
Values	Pearson correlation	1	0.509	−0.707 *
	Sig. (bilateral)		0.091	0.010
	*n*	12	12	12
Years	Pearson correlation	0.509	1	0.000
	Sig. (bilateral)	0.091		1.000
	*n*	12	12	12
Diseases	Pearson correlation	−0.707 *	0.000	1
	Sig. (bilateral)	0.010	1.000	
	*n*	12	12	12

* The correlation is significant at the 0.05 level (two-tailed).

## Data Availability

Additional data are available from the corresponding author on reasonable request.

## Abbreviations

The following abbreviations are used in this manuscript:

CHN	Chucuito Health Network
ELISA	Enzyme-Linked Immunosorbent Assay
PCA	Principal Component Analysis
